# Structural characterization of *helitrons *and their stepwise capturing of gene fragments in the maize genome

**DOI:** 10.1186/1471-2164-12-609

**Published:** 2011-12-17

**Authors:** Yongbin Dong, Xiaomin Lu, Weibin Song, Lei Shi, Mei Zhang, Hainan Zhao, Yinping Jiao, Jinsheng Lai

**Affiliations:** 1 State Key Laboratory of Agrobiotechnology and National Maize Improvement Center, Department of Plant Genetics and Breeding, China Agricultural University, Beijing, 100193, China

## Abstract

**Background:**

As a newly identified category of DNA transposon, *helitrons *have been found in a large number of eukaryotes genomes. *Helitrons *have contributed significantly to the intra-specific genome diversity in maize. Although many characteristics of *helitrons *in the maize genome have been well documented, the sequence of an intact autonomous *helitrons *has not been identified in maize. In addition, the process of gene fragment capturing during the transposition of *helitrons *has not been characterized.

**Results:**

The whole genome sequences of maize inbred line B73 were analyzed, 1,649 *helitron*-like transposons including 1,515 helAs and 134 helBs were identified. *ZmhelA1*, *ZmhelB1 *and *ZmhelB2 *all encode an open reading frame (ORF) with intact replication initiator (Rep) motif and a DNA helicase (Hel) domain, which are similar to previously reported autonomous *helitrons *in other organisms. The putative autonomous *ZmhelB1 *and *ZmhelB2 *contain an extra replication factor-a protein1 (RPA1) transposase (RPA-TPase) including three single strand DNA-binding domains (DBD)-A/-B/-C in the ORF. Over ninety percent of maize *helitrons *identified have captured gene fragments. HelAs and helBs carry 4,645 and 249 gene fragments, which yield 2,507 and 187 different genes respectively. Many *helitrons *contain mutilple terminal sequences, but only one 3'-terminal sequence had an intact "CTAG" motif. There were no significant differences in the 5'-termini sequence between the veritas terminal sequence and the pseudo sequence. *Helitrons *not only can capture fragments, but were also shown to lose internal sequences during the course of transposing.

**Conclusions:**

Three putative autonomous elements were identified, which encoded an intact Rep motif and a DNA helicase domain, suggesting that autonomous *helitrons *may exist in modern maize. The results indicate that gene fragments captured during the transposition of many *helitrons *happen in a stepwise way, with multiple gene fragments within one *helitron *resulting from several sequential transpositions. In addition, we have proposed a potential mechanism regarding how *helitrons *with multiple termini are generated.

## Background

Transposable elements (TEs) not only make up big part of genomes of higher plants, but also play an important role in promoting their genomic diversity [1,2]. *Helitrons*, a new category of DNA TEs, have recently been uncovered by the computational analysis of genomic sequences of *A. thaliana*, *O. sativa *and *C. elegans *[3]. Lacking the typical structures that are characteristic of traditional class DNA TEs, *helitrons *are difficult to be identified. However, *helitrons *have a "TC" motif on the 5'-terminus and a "CTRR" motif on the 3'-terminus; they also contain a 16-20 bp palindromic sequence, which can form a hairpin structure of 10-12 bp upstream of the 3'-terminus. In addition, they insert preferentially between adenine and thymidine nucleotides [3,4]. *Helitrons *are ubiquitous in all studied eukaryotes, such as *A. thaliana*, *C. elegans*, *D. melanogaster*, *D. rerio*, *I. tricolor*, *L. perenne*, *M. lucifugus*, *A. gambiae*, *M. Truncatula*, *N. vectensis*, *O. sativa*, *X. maculatus*, *S. bicolor*, *S. nephelus*, and *Z. mays *[3-12].

*Helitrons *constitute over 2% of the maize genome. It was estimated that there might be tens of thousands elements in maize inbred line B73 [13,14]. They could capture gene fragments and move around the genome, which leads to gene diversity between the maize inbred lines [15]. *Helitrons *have contributed the remarkable variation of haplotype in the *Bz *(bronze) genomic locus among different maize inbred lines [16,17]. Two *helitrons *containing hundreds of copies in maize inbred line B73 have been identified [13].

More *helitrons *and their capture gene fragments have been detected in maize than in *A. thaliana *and *O. sativa *[3,13,14,18,19]. Yang *et al*. [14] found that over half of the *helitrons *have contained gene fragments in the B73 genome. They could be from 28 bp to a 7.6 kb gene fragments in length, and might even include an entire gene sequence [20,21]. According to the results of Du *et al*. [13] and Yang *et al*. [14], the *helitrons *could possess zero to nine gene fragments, which came from 376 and 840 different genes. The gene fragments carried by these elements could also form chimeric genes [13,20]. ESTs of *helitron *sequences have been detected in certain maize tissues [15]. It is possible that some functional genes can be produced from the shuffling of the capture gene fragments.

The mechanism how *helitrons *capture gene fragments and how they transpose remain unknown. The replication initiator (Rep) protein motif and a DNA helicase (Hel) domain are considered to be the key protein features of rolling circle (RC) processes in bacteria [3,4,10,22]. It was postulated that *helitrons *could mobilize by the RC replication of the "copy-and-paste" model in eukaryotes [4]. Choi *et al*. [5] found a predicted autonomous element carrying *Rep*/*Hel-TPase *and *RPA*-*TPase *in *I. tricolor*, however, it contained a frameshift and a non-sense mutation. Morgante *et al*. [15] identified two sequences that contained the conserved RC-Rep motif and DNA helicase domain in two maize inbred lines. However they both are interrupted by other transposons. Du *et al*. [13] and Yang *et al*. [14] proposed that *helitrons *had amplified within the last 6 million years and could still be active in the modern maize. So far, no intact autonomous element has been discovered in maize [13,14,19].

The full genome sequence of inbred line B73 has been achieved using BAC by BAC sequencing strategy recently [23]. Du *et al*. [13,24] and Yang *et al*. [14] have developed methods for identifying *helitrons*, and mined 2,791 and 1,930 elements, respectively. They had analyzed the extensive distribution, variability and diversity of *helitrons *in the maize genome. From these studies, certain hallmarks of *helitrons *in maize have emerged, such as that they preferentially inserted near other ones, but less commonly inserted into certain gene. There were some elements with more than one 5'-termini or 3'-termini. Many *helitrons *have been shown to carry phosphatase 2C-like gene fragments.

To further understand the characteristics of *helitrons *as well as the features of their transpositions, we have again developed a set of PERL scripts to search for additional *helitrons *in the maize genome. A total of 1,649 *helitrons *have been identified including three putative autonomous elements and two *helitrons *with high copy number. Our study not only provides a detailed characterization of putative intact automomous *helitrons*, but also presents evidence to suggest that gene fragment capturing during the transposition of *helitrons *happened in a stepwise way, with multiple gene fragments within one *helitron *being the capturing the products of several sequential transpositions. We have also proposed and provided the evidence to support a mechanism regarding how multiple terminal elements are generated.

## Results

### Identification of additional *helitrons*

To obtain additional *helitrons *with high confidence, the sequences of 23 published ones [7,15,17,25-27] including twenty helAs and three helBs, were used as query sequences to search against the maize genome sequence by BLASTN. The resulting 248 candidate *helitrons *were initially identified. To further verify these candidate *helitrons*, two strategies were used. Firstly, *helitron *locating in repeated regions could be verified by BLASTN (Additional file 1, Figure S1A) [17]. Secondly, *helitrons *with multiple copies of high similarity could be verified each other by aligning their sequences together to determine their exact 5' and 3' boundaries (Additional file 1, Figure S1B). Altogether, we obtained 96 validated *helitrons *by these two methods, including eighty helAs and sixteen helBs. To further confirm these *helitrons*, we conducted PCR experiments for some selected *helitrons*. All fourteen that had successful PCR amplification showed variable in sizes of PCR products (Additional file 2, Figure S2), indicating the vacant sites and occupied sites, therefore providing final confirmation for our 96 seed *helitrons*.

Based on the terminal sequence characteristics of the 96 validated *helitrons*, a PERL script was designed to identify additional elements in the maize genome. As a result, a total of 1,649 intact elements were obtained. According to a standard previously reported [17], we divided these new elements into two different families, which including 1,515 helAs and 134 helBs (Additional file 3, 4, Table S1, S2). The size of these elements ranged from 128 bp to 20,874 bp; the average length was 6,357 bp for helA, and 4,629 bp for helB. Overall, 82.7% (1,253/1,515) of helA sequences were less than 10 kb in length. Similarly 94.8% (127/134) helB were less than 10 kb. HelAs with the length of over 10 kb (22.5%; 59/262) and all 7 helBs with the length of over 10 kb were classified as putative "autonomous" *helitrons *if they do not contain other long transposons such as retrotransposon.

HelAs had a conserved sequence of the 24 bp at the 5'-terminus and 28 bp at the 3'-terminus including palindromic structures. HelBs had conservative sequences for 28 bp and 32 bp at the 5'-terminus and 3'-terminus, respectively (Additional file 5, Figure S3). The 5'-terminus of helBs was significantly different from those of helAs.

### Putative autonomous *helitrons*

**Figure 1:**
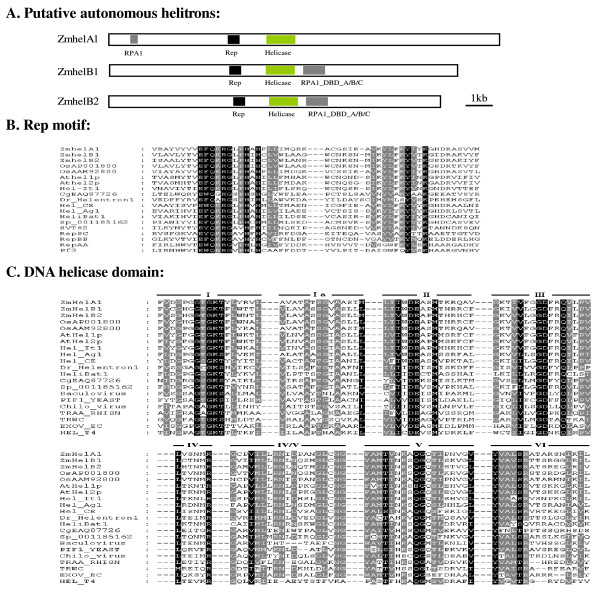
**Gene structure, the Rep protein motif and DNA helicase domain of putative autonomous *ZmhelA *and *ZmhelB***. A. A schematic diagram of putative autonomous *ZmhelA *and *ZmhelB *showing the Rep, helicases and RPA domain. B and C. Multiple sequence alignments of the Rep motif and DNA helicase domain. Sequences from other species were aligned with *ZmhelA1*, *ZmhelB1 *and *ZmhelB2*. Ce, *C. elegans*; Ag, *Anopheles gambiae*; Os, *Oryza sativa*; At, *A. thaliana*; It, *Ipomoea tricolor*; Dr, *Danio rerio*; Cg, *Chaetomium globosum*; Sp, *Strongylocentrotus purpuratus*; SVTS, *Spiroplasma plectrovirus *(AAF18311); Rep_SC, *Streptomyces cyaneus plasmid *(BAA34784); Rep_BB, *Bacillus borstelensis plasmid *(BAA07788); Rep_AA, *Actinobacillus actinomycetemcomitans plasmid *(AAC37125); Pf3, *Pseudomonas aeruginosa bacteriophage *(AAA88392); Baculovirus (NP047686); Yeast (P07271); CHilo (AAD48149); TRAA_RHISN (P55418); TRWC (S43878); EXOV_EC (P04993); HEL_T4 (P32270) [10].

**Figure 2:**
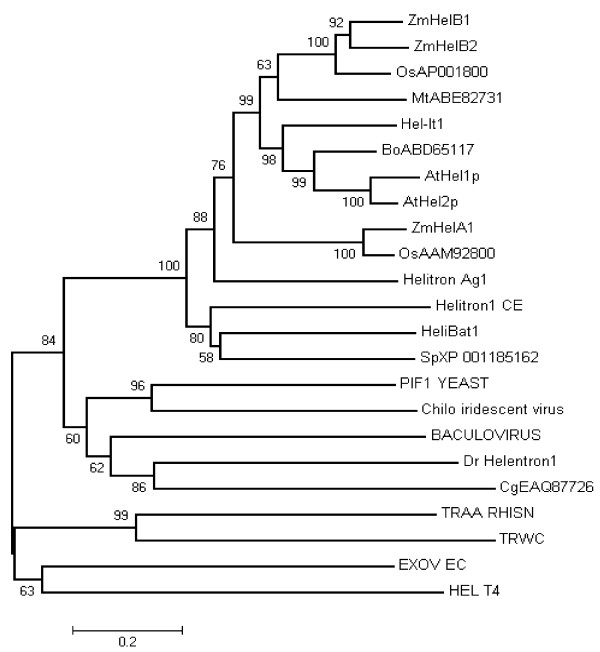
**A phylogenetic tree of DNA helicase of putative autonomous *helitrons *of maize and other species**. The phylogenetic tree was constructed by the neighbor-joining method using MEGA4 software [32] with 1,000 bootstrap replicates, the bootstrap scores < 50% were deleted. The accession numbers and names of the putative helicases of other species were abbreviated as shown in Figure 1, with the addition of the following: Bo, *Brassica oleracea *(ABD65117); Mt, *Medicago trunculata *(ABE82731) [10].

In general, the *helitrons *that encode replication initiator (Rep) motif, DNA helicase domain and a possible replication A protein 1 (RPA1)-like motif in plants, are considered as putative autonomous ones [4]. To find potential autonomous *helitrons*, all helAs sequences of over 10 kb and helBs of over 5 kb were carefully annotated. Two sequences, named *ZmhelA1 *(AC208648.2, 14,632 bp) and *ZmhelB2 *(AC212020.2, 12,217 bp) respectively were qualified as putative autonomous elements. *ZmhelA1 *and *ZmhelB2 *all contained conserved Rep motif and DNA helicase domain without frameshift (Figure 1A, B, C, Additional file 6, Table S3). Those conserved domains were reported to be essential for DNA replication and for unwinding double stranded DNA in other prokaryotic and eukaryotic species [3,5,10]. The putative autonomous *ZmhelA1 *also contained a putative RPA remnant before the Rep motif (Figure 1A), although the RPA sequence had a very low sequence homologous with that of *A. thaliana *and *O. sativa *[3]. In addition, *ZmhelA1 *also carried eight predicted gene fragments. *ZmhelB2 *possessed three putative single strand DNA-binding domains (DBD)-A/-B/-C of RPA1 following the helicase domain in the ORF (Figure 1A), which were in the same orientation as the Rep/Helicase gene. *ZmhelB2 *also carried two postulated gene fragments. Based on these structural characteristics, autonomous *helitrons *in maize could be at least divided into two types, a result that was consistent with the neighbor-joining phylogeny analysis (Figure 2).

To obtain additional putative "autonomous" elements, the RPA-like and DNA helicase of *A. thaliana *and *O. sativa *[3,5] were used to search against maize genome by TBLASTN respectively. Then the obtained sequences were extended 10 kb each in the 5'-terminus and 3'-terminus respectively. Finally, the obtained putative autonomous *helitrons *were annotated by Fgenesh (http://linux1.softberry.com/berry.phtml). As a result, five putative autonomous helBs were identified by this homolog searching approach. One of the five putative autonomous helBs, *ZmhelB1 *(AC200867.3) with the length of 12,992 bp, also encoded an intact ORF as *ZmhelB2 *with potentially functional Rep motif, a DNA helicase domain and a RPA1 motif without frameshift (Figure 1A, B, C, Additional file 6, Table S3). These two putative autonomous helBs have similar structural characteristics as that reported by Morgante *et al*. [15].

### *Helitrons *of multiple terminal sequences and of high copy number

**Figure 3:**
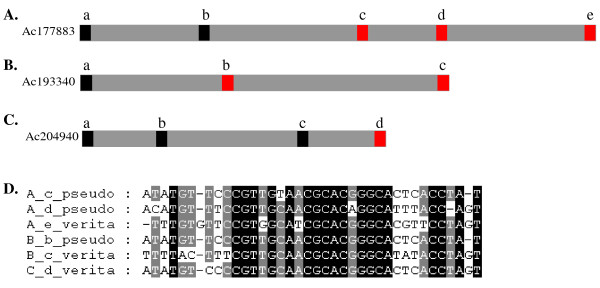
***Helitrons *of multiple termini and their sequence characteristics of 3'-termini in maize**. A, B and C. *Helitrons with *multiple termini. The black and red boxes indicate 5'-termini and 3'-termini of helA respectively. D. Alignment of the pseudo 3'-termini and the real ones in Figure 3A, B, C.

Our result showed that 28.7% of helAs had contained multiple terminal structures. We called the internal terminal sequences as the pseudo terminus (Figure 3A, B, C). Through multiple sequence alignment, we found that the real 3'-terminus of *helitrons *contained highly conserved "CTAG" motif, but not at the pseudo 3'-terminus of elements with multiple 3'-termini (Figure 3D). One hundred helAs with multiple 3'-termini were randomly sampled to analyze structure of their pseudo 3'-termini, the result showed that 99% (99/100) of the internal 3' end sequence had a pseudo 3'-terminus with no intact "CTAG" motif. However, we did not find any multiple terminal sequences in the 134 helBs.

Based on the sequence characteristics of pseudo 3'-termini that we obtained, the following consensus sequence model was defined: "CCGT[ATCG]GCA[AT]CGCACG[AG]{2}[ATCG]{6, 8}CTAT". By searching against the maize genome sequence according to the model, 662 pseudo 3'-termini sequences were obtained. Ten sequences were randomly selected from these newly identified pseudo 3'-termini, and the intact 3'-termini structures were shown within 10 kb downstream. It was ubiquitous that the pseudo 3'-termini we identified had no intact "CTAG" motif in maize. Using the same methods, we found that 17.6% of helAs also had multiple 5'-termini. However, there were no distinct differences between the pseudo 5'-terminal sequence and the true 5'-terminal one.

**Figure 4:**
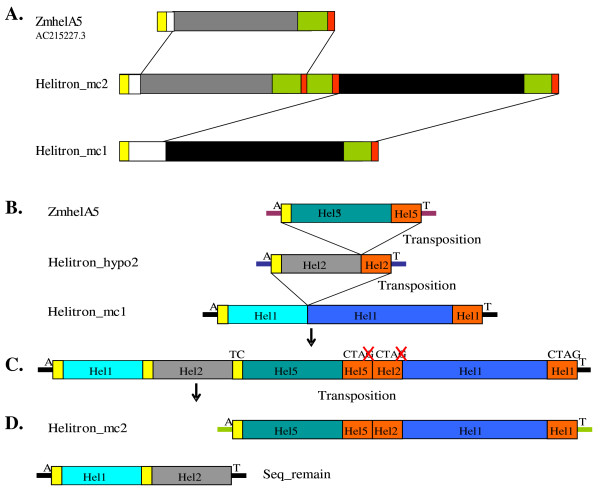
**The putative evolutionary relationship among *helitron*_*mc1*, *helitron*_*mc2 *and *ZmhelA5***. A. Structural information of three *helitrons *identified in the maize genome. The size of *helitron_mc1*, *helitron_mc2 *and *ZmhelA5 *were 1,617 bp, 2,683 bp and 1,111 bp, respectively. Sequences between two lines had identities ≥ 85%. B, C and D. The hypothesized evolutionary path for *helitron *with multiple 3' termini. B. *zmhelA5 *inserted into *helitron_hypo2 *to form new *helitron*. Then new *helitron_hypo2 *inserted into *helitron_mc1 *(hel1) to form the C state. C to D: The nested intermediate *helitron *transposed starting from the 5'-terminus of *ZmhelA5 *to generate *helitron*_*mc2 *with three 3'-termini, leaving a remnant with two 5'-termini.

*Helitrons *with many copies have been previously identified in inbred line B73 [13,14]. Here we found two additional elements with high copy number. Two of the helAs, named *helitron_mc1 *(AC186621.4, 1615 bp) and *helitron_mc2 *(AC188746.2, 2683 bp), possessed 50 and 54 copies with a high stringent criteria (coverage >95% and identities >95%), respectively. Using a more relaxed set of criteria (sequence identity >80%, size >200 bp), there were 2,450 and 5,103 copies, respectively (Table 1, Additional file 7, 8, Table S4, S5). *Helitron_mc1 *had over 85% identities in 1,300 bp of the 3'-end sequence with *helitron_mc2*. It is possible that *helitron_mc2 *have evolved from *helitron_mc1*. In addition, *helitron_mc2 *also possessed two pseudo 3'-termini structures (Figure 4).

**Table 1:** Copy numbers of *helitron*_*mc1 *and *helitron*_*mc2*.

Name	Size (bp)	Standard	Copy number
*helitron_mc1*	1,615	coverage>95% and identities>95%	50
		coverage>90% and identities>90%	88
		coverage>85% and identities>85%	332
		coverage>200 bp and identities>80%	2450
*helitron_mc2*	2,683	coverage>95% and identities>95%	54
		coverage>90% and identities>90%	89
		coverage>85% and identities>85%	94
		coverage>200 bp and identities>80%	5103

### Gene fragments captured by *helitrons*

**Figure 5:**
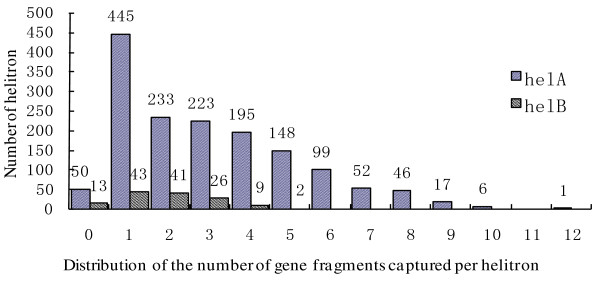
**Distribution of the number of gene fragments carried by helAs and helBs**. X-axis, the number of gene fragments; y-axis, the number of helAs and helBs.

In order to analyze the gene fragments carried by *helitrons*, all detected elements were searched against the nonredundant protein (nr) database using the BLAST program. Most of *helitrons *with a size of less than 1 kb (64.7%) did not contain any gene fragment. Most of elements with lengths from 1 kb to 2 kb (90%) had only obtained one gene segment. The number of capture gene fragments by the helAs ranged from 0 to 12, with a mean value of 3. Most helAs (82.1%) carried between 1 and 5 gene fragments. All of the helBs held no more than five gene fragments, with an average of 1.8. The majority of helBs (82%) acquired 1 to 3 gene fragments (Figure 5).

A total of 4,645 gene fragments were carried by the helAs, which encoded 2,507 proteins (Additional file 9, Table S6). There were 229 helAs that had captured a near identical fragment of phosphatase (type) 2C-like protein (ACG41393.1) [13,14], the same gene fragment found in *helitron_mc1 *and *helitron_mc2*. Different members of the phosphatase (type) 2C family protein were also captured by other helAs, such as ACF84978.1 (48 hits), AAQ06294.1 (29 hits) and ACF83293.1 (19 hits). It is possible that the phosphatase (type) 2C-like protein carried by the helAs could have been amplified previously [13].

A total of 249 gene fragments coming from 187 proteins have been captured by helBs (Additional file 10, Table S7). There were 6 helBs that contained a same gene fragment (ACG47094.1). Our results suggest that *helitrons *do not have a bias in capturing gene fragments.

### Step by step capturing of gene fragment

**Figure 6:**
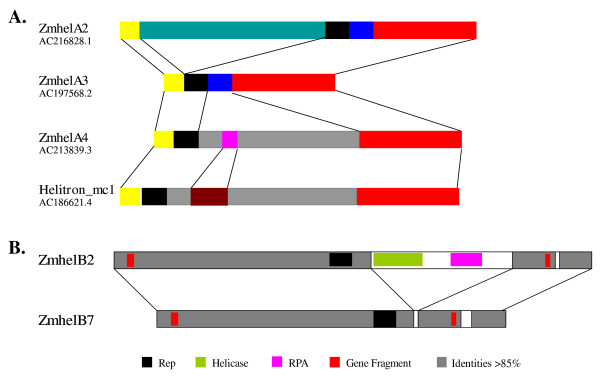
**Sequence homology of related *helitrons***. A. Fragmented sequence homology of four related *helitrons*. Accession number where the *helitrons *were identified were shown on the left, segments with the same colors have sequence identities >85%. B. Structural relationship between *ZmhelB7 *and the putative autonomous *ZmhelB2*.

Many *helitrons *have captured several gene fragments. Some of the gene fragments are apparently even from different chromosomes of the maize genome. How can a single *helitron *capture a number of gene fragments originally located in several different loci of the genome is a big puzzle thus far. Extensive sequence alignment analyses showed that there was high level but fragmented sequence homology within their captured gene fragments among a number of newly identified *helitrons*. For example, several captured gene fragments of *ZmhelA3 *(362 bp, AC197568.2) were shown to have high sequence similarity with multiple captured fragments of *ZmhelA2 *(1,728 bp, AC216828.1), *ZmhelA4 *(1,520 bp, AC213839.3), *helitron_mc1 *and *helitron_mc2 *(Figure 4A, 6A) respectively. All these four elements have near identical first 25 bp of their 5'-termini and last 30 bp of their 3'-termini. Interestingly, *ZmhelA3 *and *ZmhelA2 *have over 95% identity from 5' to 3' end, excepting one insertion in the middle for *ZmhelA2*. Therefore, *ZmhelA2 *can be explained by having captured a 1,366 bp gene fragment and having inserted into 25 bp of its 5'-termini of its ancestral element (*ZmhelA3*). In the same way, *ZmhelA4 *and *helitron_mc1 *showed high sequence similarity (more than 85%) with the 193 bp of the 3'-terminus of *ZmhelA3*. Detailed analysis indicated that, starting from an ancestral element that is missing only one internal gene fragment (shown in blue as Figure 6A) from *ZmhelA3*, the *ZmhelA4 *and *helitron_mc1 *can both be generated by capturing different gene fragments over several steps of transposition. Our result strongly suggested that the gene fragments captured by *helitrons *happened in sequential fashion, with each step of transposition likely capturing one gene fragments. In fact, such a stepwise gene capturing capacity will provide endless opportunity to shuffle gene fragments originating from all over the genome.

Z*mhelB7 *(AC186647.3, AC212020.4) might have evolved from *ZmhelB2*. *ZmhelB7 *and *ZmhelB2 *have the same terminal sequences, but the former lacked the DNA helicase domain and the replication protein A (RPA)-like fragments that were found in *ZmhelB2 *(Figure 6B). This indicated that *helitrons *could lose the internal sequence during the process of transposition in maize.

## Discussion

*Helitrons *are particularly complex in the maize genome [13,14,28]. A total of 1,649 elements were obtained based on the terminal sequence characteristics of elements in this research. Du *et al*. [13] and Yang *et al*. [14] identified 2,791 and 1,930 intact elements in the maize genome, which overlapped 52.46% and 34.45% with our result respectively (Additional file 11, 12, Table S8, S9). The differences among these three searching programs are mainly due to the parameters used in the respective perl scripts. For example, the script used by Du *et al*. [13] only aimed to identify helAs, while script used in this study is intended to cover both helAs and helBs. Additionally, Du's script and that of the current study have also differed in a number of searching criteria which leaded to a number of specific *helitrons *being identified by each script. Based on previous estimation [15], there are still a large number of *helitrons *in maize B73 genome have not been identified. Due to the unique structure of *helitrons*, it is still very difficult to unambiguously identify all these elements. With more seed *helitrons *available, a more accurate script could be generated which would drastically increase the number of elements being identified in the B73 genome.

### Putative autonomous *helitrons*

All *helitrons *that have been identified so far in the maize genome are nonautonomous [13,14]. In fact, truly autonomous elements have not been found in eukaryotic species to date. In a spontaneous *pearly-s *mutant of *I. tricolor*, Choi *et al*. [5] found that a putative autonomous *helitron *containing *Rep*/*Hel-TPase *and *RPA*-*TPase*, but it had a frameshift and a nonsense mutation. Morgante *et al*. [15] identified two sequences contained Rep motif and DNA helicase domain. However they both are interrupted by other transposons. Three putative autonomous *helitrons *found in this research have contained intact Rep motif and DNA helicase domain, the same as those found in *A*. *thaliana *and *O*. *sativa *[3]. We also detected other four helBs with the conserved Rep motif and the DNA helicase domain, however, their ORF were either having frameshift or incomplete (Additional file 6, Table S3). Although we can not confirm that these three putative autonomous *helitrons *are actually function as autonomous element at present, the presence of these three putative autonomous sequences with intact ORF in the B73 genome is strongly suggested that true autonomous *helitrons *could exist in modern maize.

*ZmhelA1 *had a putative RPA remnant before the Rep motif. *ZmhelB1 *and *ZmhelB2 *possessed an intact RPA1-like domain following the helicase domain in the same ORF respectively. Choi *et al*. [5] speculated that Rep/Helicase were ubiquitous in eukaryotes, and could play a more important role in the *helitrons *transposition than RPA1. The structural characteristics of putative autonomous elements in *A. thaliana*, *C. elegans*, *I. tricolor*, *M. lucifugus*, *O. sativa *and *Z. mays *were carefully analyzed [3,5,10,15] (Additional file 13, Table S10). The putative autonomous elements in animal only contain the conserved Rep motif and DNA helicase domain. The putative autonomous elements in plants all contain RPA-DBD-A/-B/-C before Rep motif or after helicase domain, except for the conserved Rep motif and DNA helicase domain. If *ZmhelA1 *indeed function as an autonomous element, then it would suggest that RPA1 is not an indispensable feature for *helitrons *transposition. The putative autonomous *helitrons *in plants can be divided into two types. One is RPA-DBD-A/-B/-C, following the successive Rep motif and DNA helicase domain in two different ORF respectively. The second contains the Rep motif, DNA helicase domain and RPA-DBD-A/-B/-C in their appropriate order in the same ORF.

### Generation of *helitron *with multiple termini from nested *helitrons*

Most *helitrons *in the maize genome were found to be small sizes. About 80% (1,253/1,515) helAs were between 100 bp and 10 kb in length, and 94.8% (127/134) helBs ranged from 600 bp to 10 kb in this research. Yang *et al*. [14] identified 1,930 elements, of which 95.4% (1,841/1,930) were less than 10 kb in length. The finding of *helitrons *with multiple copies suggests that they do not always capture gene fragments in the process of transposition.

There were 28.7% helAs that possessed multiple terminal structures as shown by Du *et al*. [13]. The pseudo 3'-termini sequences had damaged "CTAG" motif comparing with the real 3'-termini. We found that the pseudo 3'-termini structures were ubiquitous in maize inbred line B73. HelAs had preference to insert near to or inside other *helitrons *[14], which could have caused to form multiple terminal sequences inside them. Genomic evolution or transpositions could have caused an intact terminal structure to turn into a pseudo 3'-terminus (Figure 4B, C, D). Yang *et al*. [14] reported that *helitrons *could recognize a new 3'- or 5'-terminus site to form a new element in *A. thaliana*. Du *et al*. [13] found that the 3'-termini sequences were more variable than the 5'-termini ones.

The evolutionary pathway of *helitrons *with shared capture gene fragments can be deduced according their different combination of their capture gene fragments (Figure 6A, B). We detected two elements with multiple copies, *helitron_mc1 *and *helitron_mc2*, the latter possessed two pseudo 3'-termini structures (Figure 4). There was a high similarity in the 5'-terminal sequence of *helitron_mc2 *and *ZmhelA5 *(AC215227.3). *Helitron_mc1*, *helitron_mc2 *and *ZmhelA5 *had one, three and one fragment respectively, which are highly homologous to 193 bp of the 3'-terminus of *ZmhelA3 *(Figure 4A). According to these observations, *helitron_mc2 *might have evolved from *helitron_mc1 and ZmhelA5 *[29,30]. The detail of the hypothesized evolution path for *helitron_mc2 *is shown in Figure 4B, C, D. *ZmhelA5 *were inserted into *helitron*_*hypo2 *(a hypothesized intermediate). Then *helitron*_*hypo2 *carrying *ZmhelA5 *inserted into *helitron*_*mc1 *to form nested *heltrons*. Eventually *helitron*_*mc2 *was generated by further transposition starting from the 5'-end of *ZmhelA5 *while including the rest of three 3' ends. The intact 3' end "CTAG" motif can be mutated either before or after the generation of *helitron*_*mc2*. As there exist a large number of nested retrotransposons [31], there can be a lot of nested *helitrons *in the maize genome. The later is then served as intermediate to give rise to many *helitrons *of multiple termini seen in the B73 genome.

## Conclusions

*Helitrons *in the maize genome are variable size. When the elements transposed, they could sometimes capture gene fragments or lose their internal sequence. Gene capturing of *helitrons *can happen in a stepwise mode through sequential transpositions. Three putative autonomous *helitrons *were discovered in maize with intact replication initiator (Rep) motif and a DNA helicase (Hel) domain, similar to those identified in other species. Therefore, it is possible that active autonomous elements exist in modern maize. Our study also indicated that *helitrons *with multiple termini can be generated from nested *helitrons*.

## Methods

### Identification of new *helitrons*

We initially used 23 published *helitrons *including 20 helAs and 3 helBs [7,15,17,25-27] (downloaded from http://genomecluster.secs.oakland.edu/helitrons/). They were used as query sequences to search against the maize genome sequence by BLASTN. Searches were conducted according to the following criteria for the termini of candidate *helitrons*: 5' match coverage >25 bp, identities >70%; 3' match coverage >25 bp, identities >80%.

Two candidate elements with less than 20 kb between them were regarded as a single *helitron*. We initially obtained 248 candidate *helitrons*. A single element that had inserted into highly duplicated regions could be verified by BLASTN (Additional file 1, Figure S1A) [17]. Secondly, *helitrons *with multiple copies of high similarity are verified by aligning their sequences together to determine their exact 5' and 3' boundaries (Additional file 1, Figure S1B). Through these two methods, we finally validated 96 *helitrons*. Then primers of fourteen sequences of validated 96 elements were designed to the flanking regions upstream and downstream of the inserted element to verify the putative *helitrons*, to see the vacant sites and occupied sites displayed by different PCR bands in a set of 12 inbred lines (Additional file 2, Figure S2).

A PERL script was then written based on terminal characteristics of 96 validated elements to search against the sequence database of the inbred line B73. We applied two steps to identify *helitrons *more reliably, firstly using the following search criteria: helA 3'-end, CCCGT.{6,8}ACG[GA][GA].{6,8}CTAGT; helA 5'-end, ATC[TC][ATCG]TA[TC]TA[TCA][ATCG]{5,6}AAG; helB 3'-end, CGCC.{5,7}GGCG.{8,10}CTAGT; helB 5'-end, ATC[ATCG]{7,8}TTAAAA.

According to the search results and the validated criteria mentioned above, we searched the genomic sequences again using the stricter criteria as follows: helA 3'-end, CCGT.GCA[AT]CGCACG[GA]{2}.{7}CTAGT helA 5'-end, ATCT[ATCG]TACTAC.{5}A helB 3'-end, GCGCCC.{4}GGGCGC.{8}CTAGT helB 5'-end, ATC[TGA].{4}[TC][AC]TTAAAA A total of 1,649 intact elements were identified by this way. *Helitrons *with multiple termini were searched against the maize genome according to the following criteria, but avoiding the 3'-termini of elements that ended in a guanine base: CCGT[ATCG]GCA[AT]CGCACG[AG]{2}[ATCG]{6, 8}CTAT.

### Sequence analysis and annotation

Local BLAST software (blast-2.2.16) was used to align the sequences. A neighbor-joining phylogeny (1,000 bootstrap replications) was built for the helicases of different species by the Molecular Evolutionary Genetics Analysis (MEGA) 4.0 software [32]. CLUSTALX 2.0 software was used to align sequence. Identified *helitrons *were annotated by FGENESH (http://linux1.softberry.com/berry.phtml).

The sequences of newly identified *helitrons *(1,649) were used to blast against the nr protein sequence database in NCBI (http://www.ncbi.nlm.nih.gov/). Information about the quantity, location and annotation of capture gene fragments was obtained from the blast results.

### PCR validation of predicted *helitrons*

The twelve representative maize inbred lines, including Mo17, Huangye4, W182bn, W153r, W117, W64a, Va102, Va35, N192, B73, B37 and B68, were chosen to validate the *helitrons*. Genomic DNA samples from each line were extracted from young seedling, according to the CTAB procedure [33]. Specific primers were designed in flanking upstream and downstream sequence of known elements. PCR reactions were performed using 1ul of the obtained DNA, 2 ul 10× PCR buffer, 0.75 ul dNTPs mixture (2.5 mM each), 1ul of primer mixture (5 uM each), 0.25 ul Taq polymerase, and distilled H_2_O was added to make up the final volume of 20 ul. The PCR conditions were 1 min at 95࠷, then 35 cycles 95࠷ for 45s, x࠷ (57࠷ - 62࠷) for 45s and 72࠷ for 1 min, and a final extension of 10 min at 72࠷.

## Authors' contributions

J.L. designed the research. Y.D., X.L.,W.S. and L.S. did the data analysis. L.S., M.Z., H.Z. and Y.J wrote the PERL scripts. J.L. and Y.D. wrote the paper. All the authors have read and approved the final manuscript.

## Supplementary Material

Additional file 1 **Figure S1. Verification of candidate *helitrions***. A. Example of *helitron *inserted in repetitive sequences. B. *Helitrons *with multiple copies of high similarity can be verified each other by aligning their sequences together to determine their exact 5' and 3' boundaries. Click here for file

Additional file 2 **Figure S2. Verification of *helitrons *by PCR using 12 diversed inbred lines**. Primers were designed in flanking inserted upstream and downstream sequences of putative *helitrons*. Vacant sites and occupied sites were displayed by different band sizes of PCR products. The names of the 12 inbred lines were from 1 to 12: Mo17, Huangye4, W182bn, W153r, W117, W64a, Va102, Va35, N192, B73, B37 and B68. Click here for file

Additional file 3 **Table S1. The location of the 1515 helAs in the maize genome**. Click here for file

Additional file 4 **Table S2. The location of the 134 helBs in the maize genome**. Click here for file

Additional file 5 **Figure S3. The sequence characteristics of 5'-termini and 3'- termini of helAs and helBs**. A. 30 bp of 5'- termini of helAs; B. 40 bp of 3'-termini of helAs; C. 30 bp of 5'-termini of helBs; D. 40 bp of 3'-termini of helBs. Click here for file

Additional file 6 **Table S3. The putative autonomous *helitrons***. The location of the putative autonomous *helitrons *in the maize genome. Click here for file

Additional file 7 **Table S4. The location of *helitron*_*mc1 *in the maize genome**. Click here for file

Additional file 8 **Table S5. The location of *helitron*_*mc2 *in the maize genome**. Click here for file

Additional file 9 **Table S6. Gene fragments carried by helAs**. Annotated protein of gene fragments carried by helAs. Click here for file

Additional file 10 **Table S7. Gene fragments carried by helBs**. Annotated protein of gene fragments carried by helBs. Click here for file

Additional file 11 **Table S8. Cross-referencing of *helitrons *between our result and Yang *et al*.'s result**. Click here for file

Additional file 12 **Table S9. Cross-referencing of *helitrons *between our result and Du *et al*.'s result**. Click here for file

Additional file 13 **Table S10. The characteristic of autonomous *helitrons *in eukaryotes**. "----" indicated RPA, following the successive Rep motif and DNA helicase domain in two different ORF respectively. "--" indicated Rep motif, DNA helicase domain and RPA-DBD-A/-B/-C in their appropriate order in the same ORF. Click here for file
