# Supplementary figures and images for: Structural characterization of helitrons and their stepwise capturing of gene fragments in the maize genome
Source: BMC Genomics. 2011 Dec 17;12:609. doi: 10.1186/1471-2164-12-609 (PMC3288121; doi:10.1186/1471-2164-12-609)

## Slide 1
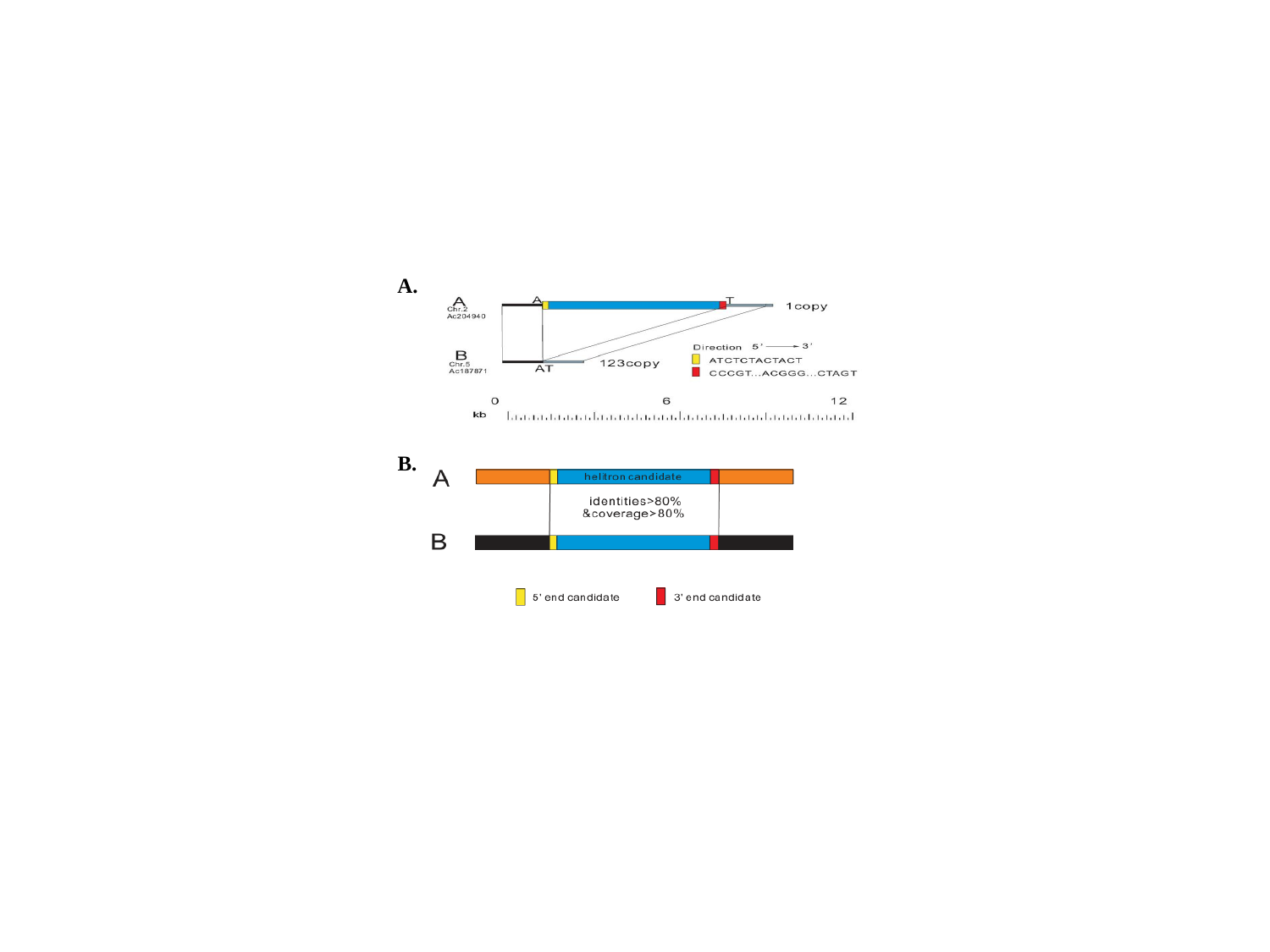

A.
B.

Supplement: Additional file 1 — Figure S1. Verification of candidate helitrions. A. Example of helitron inserted in repetitive sequences. B. Helitrons with multiple copies of high similarity can be verified each other by aligning their sequences together to determine their exact 5' and 3' boundaries. [file 1471-2164-12-609-S1.PPT]

## Slide 1
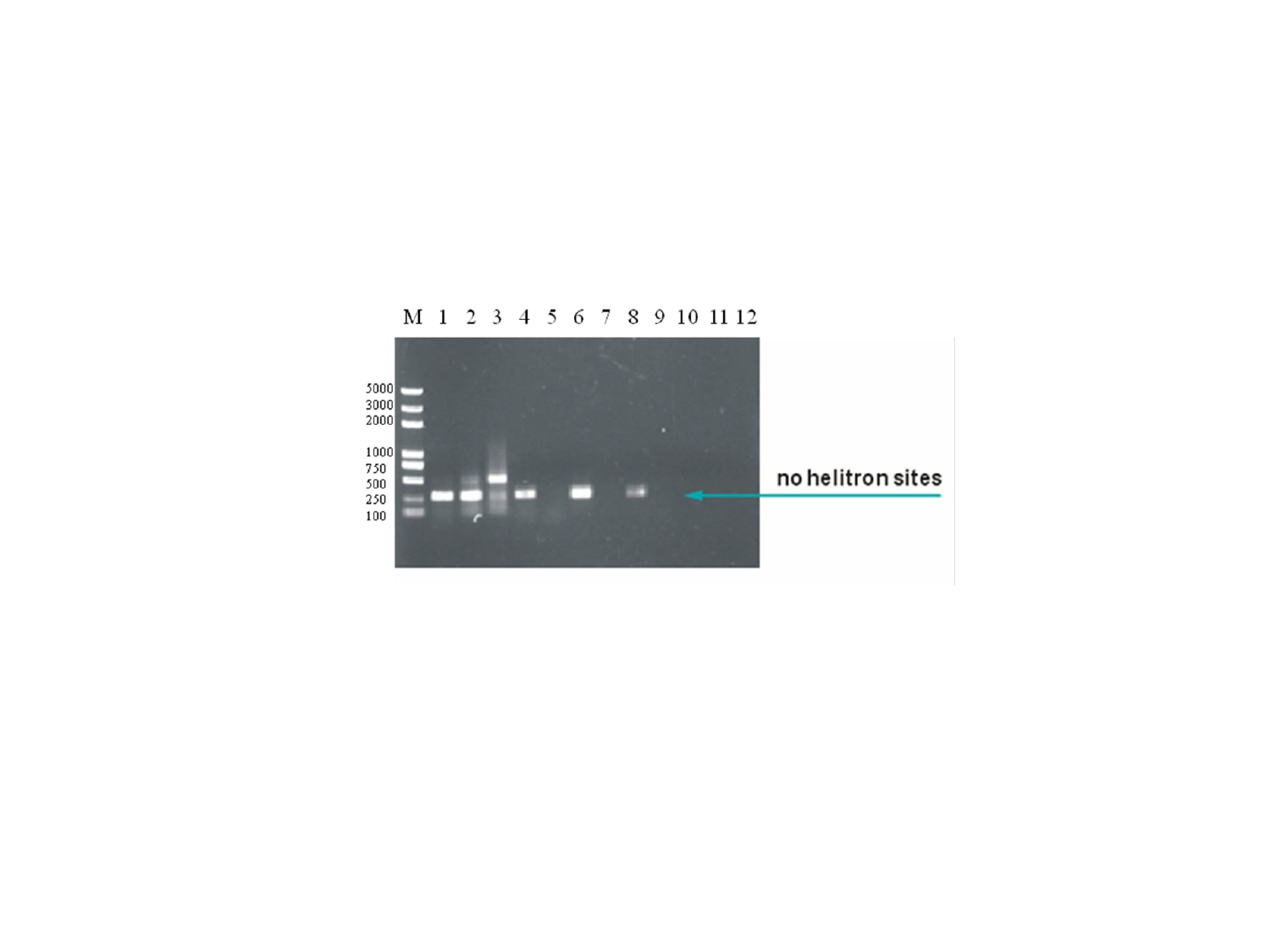

Supplement: Additional file 2 — Figure S2. Verification of helitrons by PCR using 12 diversed inbred lines. Primers were designed in flanking inserted upstream and downstream sequences of putative helitrons. Vacant sites and occupied sites were displayed by different band sizes of PCR products. The names of the 12 inbred lines were from 1 to 12: Mo17, Huangye4, W182bn, W153r, W117, W64a, Va102, Va35, N192, B73, B37 and B68. [file 1471-2164-12-609-S2.PPT]

## Slide 1
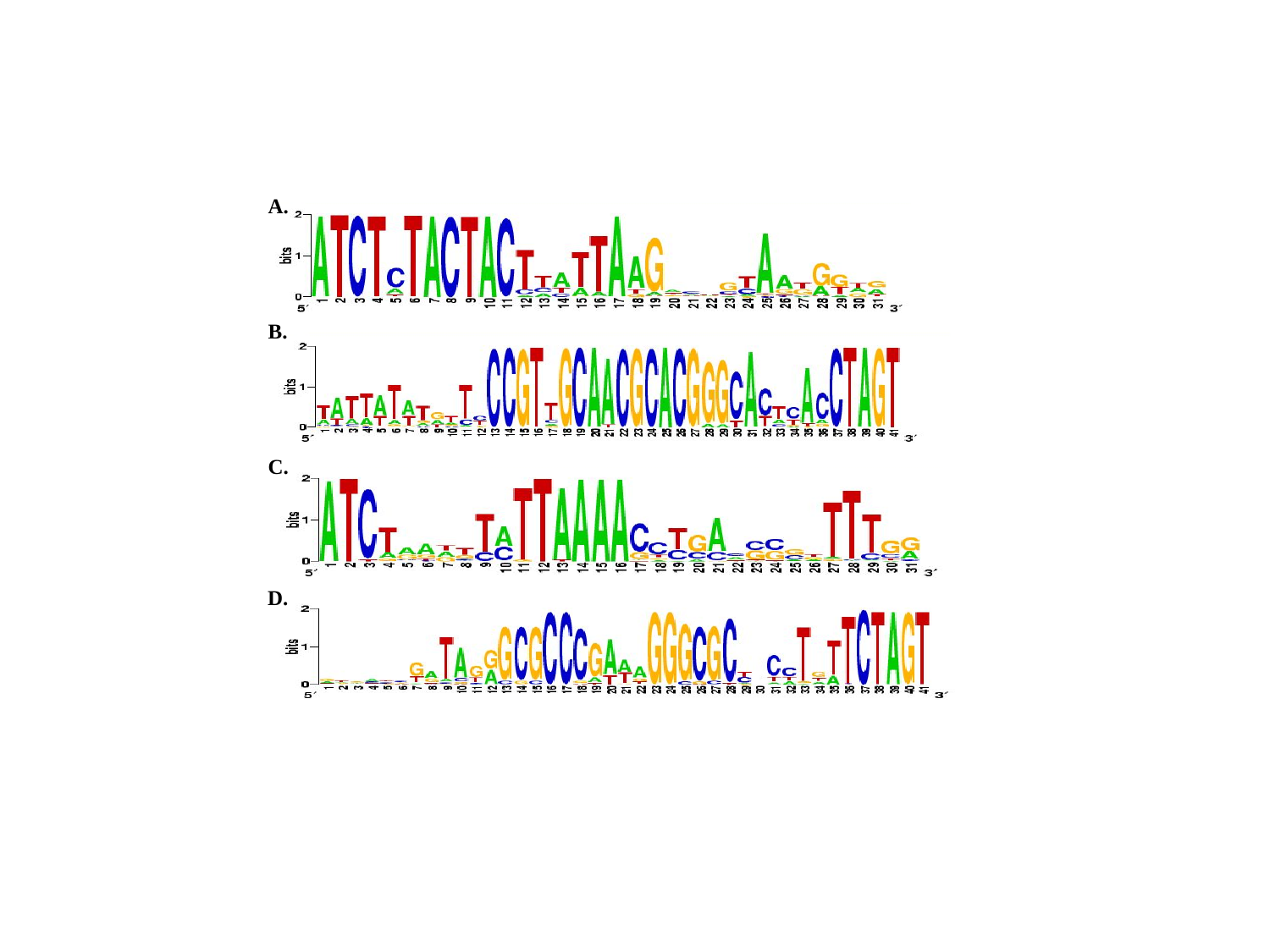

A.
B.
C.
D.

Supplement: Additional file 5 — Figure S3. The sequence characteristics of 5'-termini and 3'- termini of helAs and helBs. A. 30 bp of 5'- termini of helAs; B. 40 bp of 3'-termini of helAs; C. 30 bp of 5'-termini of helBs; D. 40 bp of 3'-termini of helBs. [file 1471-2164-12-609-S5.PPT]
